# Recurrent deletions of the X chromosome linked CNV64, CNV67, and CNV69 shows geographic differences across China and no association with idiopathic infertility in men

**DOI:** 10.1371/journal.pone.0185084

**Published:** 2017-09-21

**Authors:** Xiulan Ma, Martin Kuete, Xiuli Gu, Hui Zhou, Chengliang Xiong, Honggang Li

**Affiliations:** 1 Family Planning Research Institute/Center of Reproductive Medicine, Tongji Medical College, Huazhong University of Science and Technology, Wuhan, Hubei, China; 2 University of Montagnes, Faculty of Health Sciences, Bangante, Cameroon; 3 Tongji Reproductive Medicine Hospital, Wuhan, China; Nanjing Medical University, CHINA

## Abstract

A recent study found that three recurrent deletions of X chromosome linked copy number variations (CNVs), CNV64, CNV67 and CNV69 were associated with idiopathic male infertility in Spanish and Italian populations, especially CNV67 resembling the azoospermia factor deletions. That merits further investigations among different populations. This study was conducted to examine the prevalence of the three CNVs deletions and their associations with idiopathic male infertility in Chinese Han population. The present study included a large population of 1550 Chinese Han subjects recruited between 2014 and 2016. In total, 714 infertile participants were diagnosed as idiopathic infertility with different conditions (288 with non-obstructive azoospermia, 210 oligozoospermia and 216 asthenospermia) and 836 fertile participants (vasectomized men). The fertile participants were recruited from the representative areas: the north (Hebei and Shanxi), center (Hubei and Jiangsu), and south (Guangdong) of China. All patients were recruited from Hubei province. A multiplex PCR system was established to screen the deletion of the three CNVs, and deletion was confirmed by general PCR. Similar rates of these deletions were observed in infertile men and fertile participants (Hubei), and among the different conditions of infertility. Moreover, CNV64 and CNV67 map distribution geographically differed across China. The three CNVs in fertile groups of other regions were similar, except for Guangdong. No association between the three CNVs deletions and idiopathic male infertility was observed. CNV67 is rare in central China, albeit large sample size study for confirmation is warranted. It seems that the association between these CNVs deletions and idiopathic male infertility is ethnic dependent. There is still need to screen the CNVs deletions in other ethnicities. We suggested to consider the stratification patterns and geographic differences when prescribing CNVs deletions screening as a test in male infertility.

## Introduction

Male infertility afflicts up to 7% of men of reproductive age, whereas the etiology of more than half of the cases remains unidentified. This leads to the prevalent diagnosis of idiopathic male infertility [1]. Genetic causes may be the most common etiology of unexplained male infertility, particularly in patients with spermatogenic impairments, including non-obstructive azoospermia (NOA), oligozoospermia and asthenozoospermia or the combination of the latter two [1,2]. The proven genetic causes mainly refer to karyotype abnormalities and Y chromosome microdeletions (AZF deletions) [3]. Recent Genome Wide Associate Studies (GWAS) have shown some susceptibility loci that may explain some of the unclear genetic bases of idiopathic male infertility[4–6]. Nevertheless, they are only a tip of the iceberg and large portions of genetic causes remain unknown [1].

Among the numerous genetic factors that may be the underlying etiology of idiopathic male infertility, much light has been shed on CNVs over the past decade [7–11]. CNVs have been found to be responsible for a wide range of human diseases [12]. They are responsible for 15% of the neuro-developmental disorders, cardiac abnormalities, and other abnormalities.[13]

CNVs on sex chromosomes are more likely to play key roles in germ cell development [14–17]. The role of AZF microdeletions of the Y chromosome is well characterized in male infertility while the role CNVs of the X chromosome is less established in this field [18]. In recent years, several previous studies have been focused on the association between X-linked CNVs and male infertility because of the single copy of X chromosome for men [8,9,19–21]. Since Tüttelmann had reported the first CNV in male infertility [10], many risk loci were discovered on both sex chromosomes and autosomes [8,9,22–24]. Recently, Krausz et *al*. identified three X-linked recurrent deletions, CNV64 (Xq27.3), CNV67 (Xq28) and CNV69 (Xq28), which were significantly associated with male infertility in Caucasians. Particularly, CNV67, which resembles AZF deletions on the Y chromosome was considered to be specific to infertile male patients with potential clinical application [25].

However, the existing studies regarding the three CNV recurrent were only carried out in Caucasians (in South and East European cohort) [25,26], and the effects of ethnicity on the prevalence and pathogenicity of the recurrent deletions still need to be explored. Sometimes, ethnic differences exist in the genetic etiology of male infertility. For example, the prevalence of gr/gr deletion in infertile men is ethnic and geography depended. This deletion is significantly associated with male infertility risk in Caucasian and Mongolian populations, but not in mixed ethnic groups and other regions. It is also demonstrated that gr /gr deletions are associated with the haplogroup D2b and Q1, that are usually occurred in China and Japanese, not associated with male infertility[27]. Similarly, mutations in fibrosis transmembrane conductance regulator (CFTR) in congenital bilateral absence of the vas deferens were found to vary significantly between the Caucasian and non-Caucasian groups. F508delt allele is more prevalent in Caucasian than in Chinese populations[28].

Herein, the three X-linked recurrent deletions, CNV64, CNV67 and CNV69 were tested among Chinese Han population. We hypothesized that the three CNVs might be associated with idiopathic male infertility in different subtypes of abnormal spermatogenesis, just like the AZF deletions do. Idiopathic infertile men of the present study were subdivided into NOA, oligozoospermia and asthenozoospermia subgroups.

## Materials and methods

This study was carried out in the Family Planning Research Institute/Center of Reproductive Medicine at Tongji Medical College of Huazhong University of Science and Technology from 2014 to 2016. All semen analyses were performed in the laboratory of Tongji Reproductive Medicine Hospital, Wuhan, China.

### Study population

Five provinces of China, which represent the Chinese Han population, with Guangdong province in the south, Hubei and Jiangsu in the centre, and Shanxi and Hebei in the north of China, were included in the present study. Totally 1550 men were recruited: (i) the fertile group, consisted of 836 vasectomized men from five provinces, representing the Chinese Han population of the south (Guangdong 248); the center (241 from Hubei and 102 from Jiangsu) and the north (151 from Shanxi and 94 from Hebei) (S1 Fig), and (ii) infertile groups: subjects with idiopathic infertility from Hubei Province, including 288 patients with NOA, 210 with oligozoospermia (with / without asthenospermia and teratozoospermia) and 216 with asthenospermia only (with normal sperm concentration). All infertile men have primary infertility and their ages ranged from 24 to 46 years at an average of 30.6, while ages of the fertile group range from 40 to 80 years at an average of 58.2. Notably, the vasectomized subjects were fertile men who had fathered at least two children naturally.

The patients with idiopathic infertility based on a comprehensive andrological examinations including medical history, physical examination, semen analysis, karyotype analysis (patients with azoospermia or oligozoospermia) and Y choromosome microdeletions screening (patients with sperm density of ≤5 million/mL) were included in this study. While, those with known infertility causes, such as cryptorchidism, obstructive azoospermia, varicocele, infection, iatrogenic infertility, hypogonadotrophic hypogonadism, karyotype anomalies, Y chromosome microdeletions and patients of non-Han ethics origin were excluded (S2 Fig).

Three separate semen samples of each patient at the corresponding times were analyzed to confirm the diagnoses of azoospermia, oligozoospermia and asthenospermia according to the 2010 World Health Organization (WHO) guidelines [29]. And their female partners did not show apparent reproductive pathology.

This study was approved by the Institutional Review Board of Tongji Medical College, Huazhong University of Science and Technology (IRB Approval File No.[2014] 09) in accordance with the Declaration of Helsinki. Informed and written consents were obtained from all participants.

### Extraction of peripheral blood genomic DNA

Blood sample of each subject was collected in a tube with ethylene diamine teraacetic acid (EDTA) and stored under -80°C. The genomic DNA was extracted using the QIAamp Bood Kit (Qiagen, Hilden, Germany) according to the manufacturer’s handbook. DNA purity and concentration were tested by ultraviolet spectrophotometer (Biometra, Göttingen, Germany) at 260 nm and 280 nm.

### Deletions screening and validation

#### Deletions screening

Multiplex PCR assay was performed with Sequence Tag Site (STS) of CNV64, CNV67 and CNV69 in the first step of screening. And PCR+/- protocol was also designed to confirm the deletions. The sets of primers for screening and validation were the same as the previous study[25], except that the reverse primer for CNV69 deletion screening was changed into 5’- ACCGTTATATGATATCCCTCCT-3’, which is located about 80bp down the previous primer. Thus the PCR amplicons for the screening of CNV64, CNV 67, and CNV69 deletions were 193bp, 146bp, and 288bp, respectively, and can be easily separated and observed by routine agarose gel electrophoresis (S3 Fig).

The multiplex PCR for screening was performed in a final volume of 20 μl mixture containing 2.8 μl DNA of each sample (the concentration was 70 ng μl^-1^), 2 μl of a dNTPs mixture (0.2 mM each dNTP), 2 μl of 10× PCR buffer, 8 μl of the three pairs specific primers mixture (the concentrations of the three pairs primers were 1.2 μM, 1.0 μM, 1.8 μM respectively), and 0.2 μl (5.0 U μl^-1^) of DNA Polymerase (TaKaRa Dalian, China). Amplification consisted of an initial denaturation at 94°C for 5 min, 35 cycles of denaturation at 95°C for 20 s, annealing at 57°C for 30 s, and extension at 72°C for 20 s; and the final extension at 72°C for 5 min. Negative control reactions without any template DNA and the positive control reactions with the female DNA template were carried out simultaneously.

#### Deletions validation

General PCR was performed to confirm the deletions by using the screening primers separately, and to validate the deletions using the validation primer sets in lowering anneal temperature from 57°C to 52°C under the same standard conditions. The reaction system was 20 μl, containing 2 μl of the DNA template, and 2 μl of the pair of primers with concentration of 5 μM, and concentrations of the other reagents were the same as the multiplex PCR. In addition, negative control reactions without any template DNA and the positive control reactions with the female DNA template were carried out simultaneously. The PCR products were detected by 1.8% agarose gel electrophoresis. The above general PCR and the second confirmation step were repeated under strict conditions several times.

#### Statistical analysis

Statistical analysis was performed using Statistical Package for Social Sciences (SPSS Inc., Chicago, IL, USA) version 20.0. The differences between the variables were determined by Chi-square test or a Fisher exact test, and then corrected by the Bonferroni test for multiple testing. Statistical significance was set at the *P*-value < 0.05.

## Results

### The multiplex PCR system

The multiplex PCR was designed to easily detect the three recurrent deletions at the same time in one amplification system. The amplicons were all strong and clearly separated using the general agarose gel electrophoresis. No obvious nonspecific amplication which may interfere the electrophoresis result was observed. Representative results of general PCR and multiplex PCR are shown in S3 Fig.

Then we applied this multiple PCR system to screen every deletion. All cases with deletion were confirmed by the general PCR targeting the deleted region in the multiplex PCR. Firstly, the screening of the recurrent deletion of CNV64, CNV67, and CNV69 was carried out among the 836 fertile men. Secondly, we screened the three deletions in patients with NOA, oligozoospermia and asthenospermia respectively.

### CNVs deletion in north, central and south China in fertile men

The north, south, and center of China were surrogated by fertile individuals from Hebei and Shanxi, Guangdong, Jiangsu and Hubei respectively. CNV64 and CNV67 deletions were significantly different between the south China and other regions. As shown in Table 1, the total CNV64 deletion rate was 3.71% (31/836) and 5.31% in north China (Beijing 5/94, Shanxi 8/151), 4.96% in central China (Hubei 12/241, Jiangsu 5/102), 0.40% in south China (Guangdong 1/248). The rate of CNV64 deletion in Guangdong was significantly lower than in other regions (*P* = 0.004), while its deletion in other four provinces was quite similar. CNV67 deletion rate in Guangdong population was about 3.23%, significantly higher than in other regions (*P* = 0.01), of which only one case with the deletion was found in Shanxi population. Regarding CNV69, only one deletion was detected (in Hubei province).

**Table 1:** Geographic distribution of the three CNVs across China.

Region	N	CNV64 deletion	Frequency	P	CNV67 deletion	Frequency	P	CNV69 deletion	Frequency	P
***North***	***245***	***13***	***5*.*31*%**	0.004*	***1***	***0*.*41*%**	0.001*	***0***	***0*.*00*%**	1.000
Hebei	94	5	5.32%		0	0.00%		0	0.00%	
Shanxi	151	8	5.30%		1	0.66%		0	0.00%	
***Central***	***343***	***17***	***4*.*96*%**		***0***	***0*.*00*%**		***1***	***0*.*29*%**	
Hubei	241	12	4.98%		0	0.00%		1	0.41%	
Jiangsu	102	5	4.90%		0	0.00%		0	0.00%	
***South***	***248***	***1***	***0*.*40*%**		***8***	***3*.*23*%**		***0***	***0*.*00*%**	
Guangdong	248	1	0.40%		8	3.23%		0	0.00%	
**Total**	**836**	**31**	**3.71%**		**9**	**1.08%**		**1**	**0.12%**	

N = number; Freq = frequency; P = P-values. The P-values are tested by the Chi-square test and Fisher’s test and was corrected by the Bonferroni test;CNVs, copy number variations.

*P<0.05 significant association.

#### CNVs deletion in patients with idiopathic infertility in Hubei

CNV64 deletion rates in patients with NOA, oligozoospermia and asthenospermia were 4.86%, 5.24%, and 5.56% respectively. The prevalent rates were similar to the fertile men originated from central China (4.96%). Considering that infertile subjects were recruited from Hubei, a more strict comparison between the different conditions of infertile men and fertile men originated from the same region showed no significant difference (Table 2).

**Table 2:** The three CNVs in different conditions of male infertility.

Conditions	N	CNV64			CNV67			CNV69		
		Deletion	%	P	Deletion	%	P	Deletion	%	P
NOA	288	14	4.86%	0.950	0	0.0%	/	1	0.35%	1.000
Oligozoospermia	210	11	5.24%	0.901	1	0.48%	0.466	0	0.0%	1.000
Asthenospermia	216	12	5.56%	0.783	1	0.46%	0.473	0	0.0%	1.000
Fertile men	241	12	4.98%	/	0	0.0%	/	1	0.41%	/

N = number; % = percentage; P = P-value; NA = not applicable. The P-value was calculated by comparing different conditions of male infertility with the fertile men; CNVs, copy number variations; NOA, non-obstructive azoospermia.

Overall, CNV69 deletion was rare in different conditions of infertile men and fertile men (one in NOA group, one in fertile group). CNV67 deletion was also scare, and only two cases were observed in 714 patients (one in oligozoospermia and one in asthenospermia).

## Discussion

A recent study identified three recurrent deletions of X chromosome-linked CNVs (CNV64, CNV67 and CNV69) associated with idiopathic male infertility in South European Caucasians [9,25]. To note, CNV67 deletion was only found in infertile patients, which seemed to standing much promising in future clinical application like the AZF deletions on the Y chromosome [25]. However, further investigations are needed for the following reasons. First, the three deletions among different ethnicities other than Caucasians remain uncharacterized. Second, the characterization of these CNVs in different infertile subgroups deserves further analysis. The present study is the first Asian study to assess the X-linked CNVs among Chinese Han population, and included a large number of idiopathic cases with three subtypes. Moreover, we rigorously divided the subjects with diverse sociodemographic patterns distributed geographically across China’s territory. Since the Chinese Han populations are divided into North, Central and South according to the HLA (Human Leukocyte Antigens Allele) of Chinese Han Population [30], subjects in the current study were accordingly distributed in China (S1 Fig). Overall, no association existed between the three CNVs and idiopathic azoospermia, oligozoospermia, and asthenospermia among Chinese Han population. More importantly, we found that there was a significant difference of CNV64 and CNV67 deletions geographically among fertile populations in China.

Our results in Chinese Han population differ from the previous study of South European Caucasians. The latter study found that CNV64 and CNV69 deletions were prevalent among patients, and CNV67 deletion was patient specific [25]. In Chinese Han population, CNV67 deletion did not show significant difference between patient and control group in Hubei. But CNV67 deletion was commonly found among fertile men in South China and very rare in other regions. In contrast, CNV67 deletion rate of fertile men in South China (3.23%) was even higher than that of the total patient group (1.12%) in the South European study [25]. However, CNV67 deletion was not observed at all in the East European cohort [26]. Therefore, we concluded that CNV67 deletion is prevalent among the Guangdong fertile population and quite scare in other regions. No obvious association with idiopathic male infertility of CNV67 deletion could be justified by the relatively small sample size of the fertile group of Hubei in our study. A large population studies is needed to confirm this finding on CNV67 deletion.

Concerning the CNV64 deletion, no significant difference existed between the fertile, total patients or subtype groups from Hubei province. Similarly, no expected prevalence differences existed among patients with NOA, oligozoospermia and asthenospermia. And our study also found that CNV64 deletion frequency in NOA (4.86%) and oligozoospermia (5.24%) were similar to that in the South European cohort (4.29% in NOA, 5.40% in oligozoospermia) respectively. However, the deletion rate of patients with asthenospermia (with normal sperm concentration) in our study (5.56%) was close to that of the total patient group from South European study (5.74%). On the other hand, the rate of CNV64 deletion of our fertile group was much lower than theirs. The CNV64 prevalence in Hubei (both fertile and infertile) was higher than that of the patient group (3.71%) and lower than the control group (11.45%) of the East European study [23]. Definitely, CNV64 deletion in Guangdong fertile population was extremely lower than in fertile group of other regions. Thus, our finding cannot substantiate that CNV64 deletion is prevalent in patients with idiopathic infertility. Therefore, we preliminarily concluded that CNV64 and CNV67 deletions are geographically distributed in Chinese Han population.

With regard to CNV69, deletion rates in both the fertile and patient groups were very low including the Guangdong population. Additionally, the rates were also much lower when we compared them with that of the fertile and infertile group in the previous European studies [25]. So, we deemed that the CNV69 deletion did not appear to be associated with idiopathic male infertility in Han Chinese population.

Moreover, the deletion pattern of CNV67 which resembles that of AZFc deletions was not found in our result of NOA and oligozoospermia [2,31]. We speculated on the following explanation. As mentioned in the previous study, some regulatory elements and genes were described within or nearby the three CNVs deletion regions [25]. Perhaps the CNVs deletions disrupted the spermatogenesis of Caucasian population but not so obvious in Chinese Han population. Because thousands of genes with combined effects are involved in spermatogenesis, we speculated that multiple CNVs have a synergistic effect on spermatogenesis in Chinese population or this effect is too slight to illuminate the association between a single CNV and the disease. Though, this assumption warrants further studies in large population or other ethnicities to be confirmed.

Both CNV64 and CNV67 deletion rates in Guangdong population were significantly different (lower or higher) compared with other groups. These geographic differences were also observed in GWAS [32,33]. Moreover, recent studies on Chinese population genetics revealed that some of human leukocyte antigen (HLA) and Genome-wide SNP variations of the south China were distinct from that of the north and central China [32–35]. Their findings indicated that significant genetic differences existed among South-North Han Chinese subpopulations. In addition, the authors consider that Chinese nation consists of two distinct subpopulations, the north and the south. And these two subpopulations originated from two different ancestors (the studies found that north and central Chinese population derived from the same ancestors and have similar gene backgrounds). It seems that these evidences may help to explain the geographic differences of CNV64 and CNV67.

Since Chinese population makes up one-fifth worldwide population, and Han ethnic constitutes about 91.51% of Chinese population, caution must be paid on genetic study design, especially when associations are interpreted. Previous studies have shown that stratification in the Chinese Han population and gene background varied significantly [12,33]. Therefore, due to the large population of China mainly dominated by Han ethnicity, we suggest to consider in future the stratification patterns and geographic differences when screening other genetic variations in Chinese patients notably from Guangdong province as well as in other parts of world.

In summary, our study is the first to screen the three X-linked CNVs among a large Asian population and we introduced an easy way to detect the three deletions at the same time. The stratification of subjects geographically represented the large and vast areas of China (north, center and south). It is the first time to provide substantial evidence of disproportional distribution of the three X-linked CNVs in China’s population. Results in South European and East European (both population are Caucasians) also differ. This study calls for caution to be paid on factors like ethnicity and area of patients when screening their X-linked CNVs deletion for idiopathic infertility causes detection in the future. The limitation of our study is patients with male infertility from different regions were not investigated. And the characteristics of these CNVs are unknown.

## Conclusion

In conclusion, the three X-linked CNVs recurrent deletions were not associated with idiopathic male infertility in Han population of central China. However, we find the disproportional distribution of CNV64 and CNV67 deletions in China.

## Supporting information

S1 Fig Geographic distribution of the study population. (TIF)

S2 Fig Flow diagram of patients inclusion/exclusion. (PDF)

S3 Fig Representative result of multiplex PCR detected by agarose gel electrophoresis. PC, positive control; NTC, no template control; M, 100 bp ladder marker, and the brightest band is 500 bp; 1 and 2, the representative CNV64 deletion; 3 and 4, the representative CNV67 deletion; 5 and 6, the representative CNV67 deletion.
(TIF)
