# Supplementary figures and images for: Recurrent deletions of the X chromosome linked CNV64, CNV67, and CNV69 shows geographic differences across China and no association with idiopathic infertility in men
Source: PLoS One. 2017 Sep 21;12(9):e0185084. doi: 10.1371/journal.pone.0185084 (PMC5608304; doi:10.1371/journal.pone.0185084)

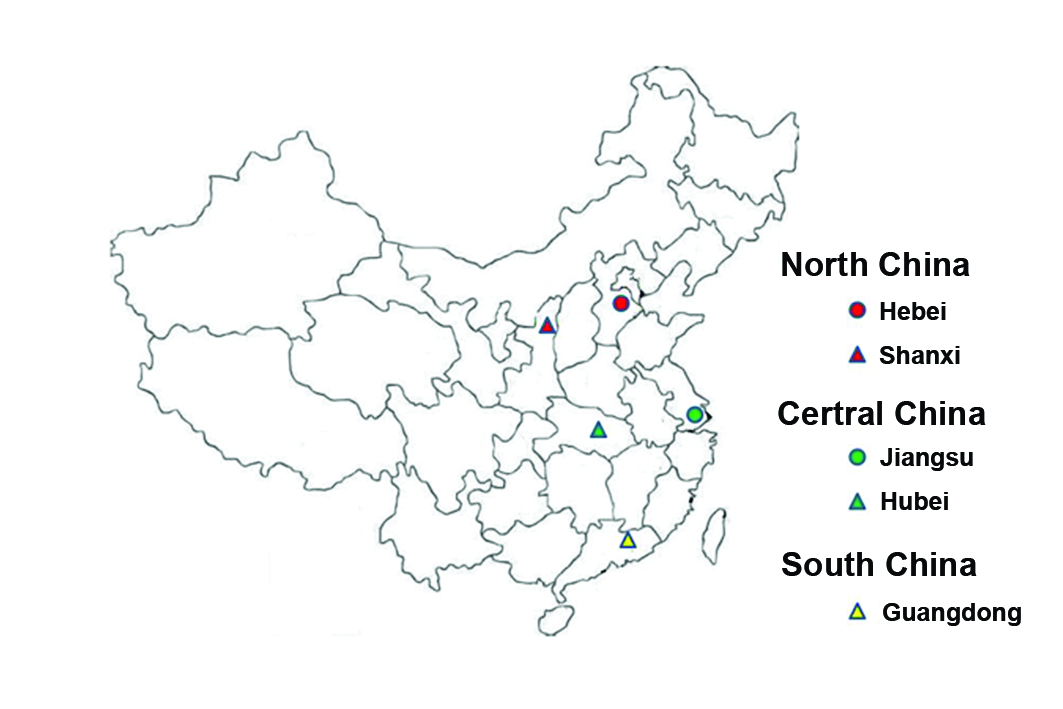

Supplement: S1 Fig — (TIF) [file pone.0185084.s001.tif]

## Flow diagram of patients inclusion/exclusion

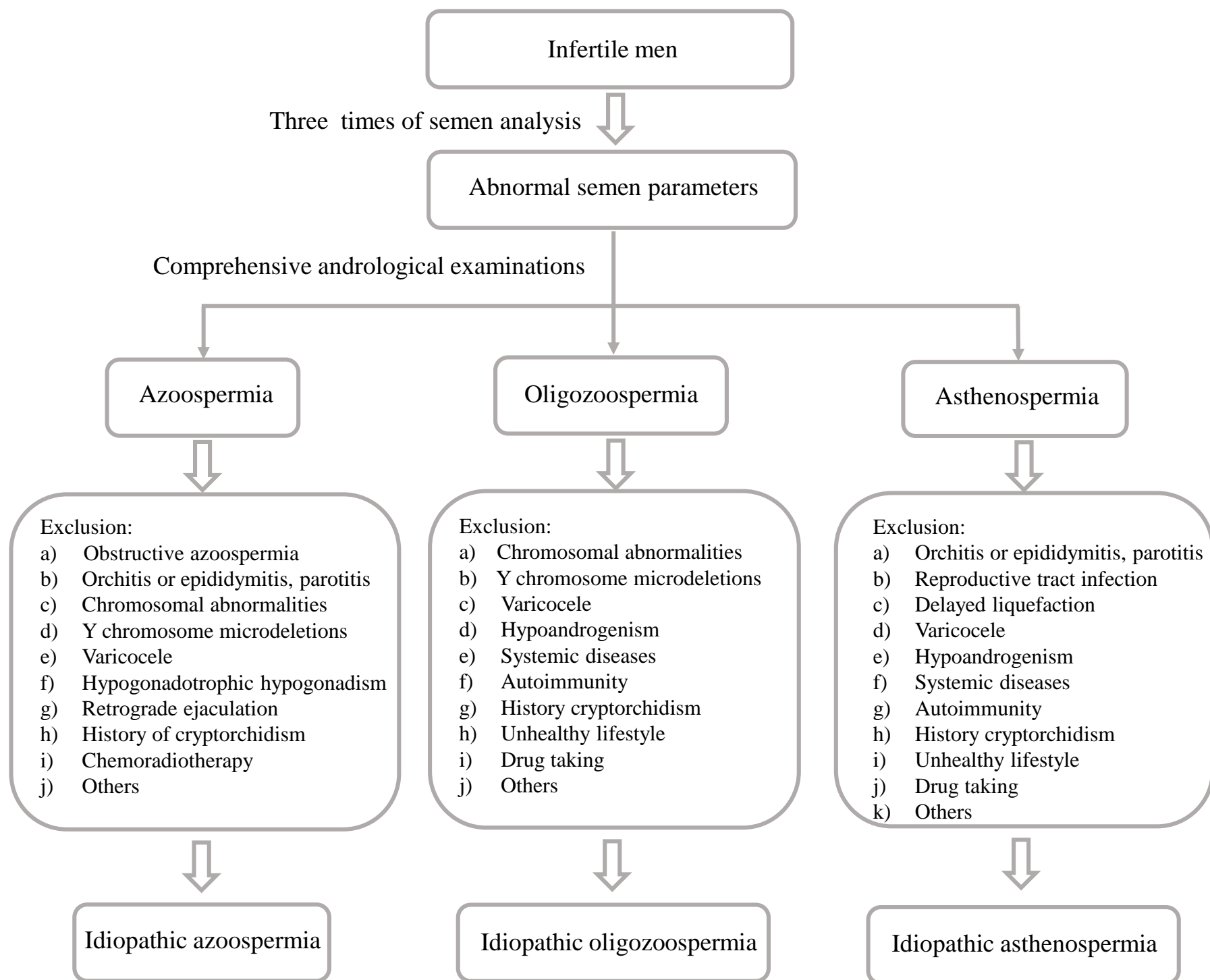

Supplement: S2 Fig — (PDF) [file pone.0185084.s002.pdf]

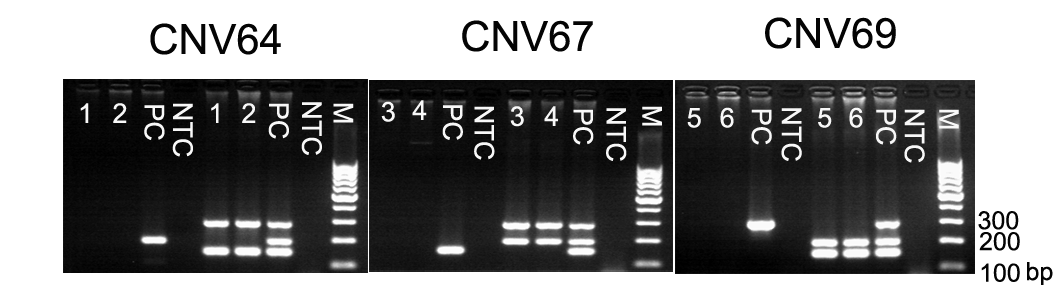

Supplement: S3 Fig — PC, positive control; NTC, no template control; M, 100 bp ladder marker, and the brightest band is 500 bp; 1 and 2, the representative CNV64 deletion; 3 and 4, the representative CNV67 deletion; 5 and 6, the representative CNV67 deletion. (TIF) [file pone.0185084.s003.tif]
